# Effects of Coenzyme Q10 Supplementation on Glycemic Control Biomarkers: An Umbrella Review of Meta‐Analyses of Randomised Controlled Trials

**DOI:** 10.1002/edm2.70182

**Published:** 2026-03-20

**Authors:** Vali Musazadeh, Maryam Falahatzadeh, Mahsa Mahmoudinezhad, Erfan Shahhooseini, Mehrdad Jamali, Pedram Pam, Farzad Shidfar

**Affiliations:** ^1^ Student Research Committee, School of Public Health Iran University of Medical Sciences Tehran Iran; ^2^ Department of Nutrition School of Public Health, Iran University of Medical Sciences Tehran Iran; ^3^ Department of Pharmacy Shiraz University of Medical Sciences Shiraz Iran; ^4^ Student Research Committee Urmia University of Medical Sciences Urmia Iran; ^5^ Department of Nutrition, School of Medicine Urmia University of Medical Sciences Urmia Iran; ^6^ Tehran University of Medical Sciences Tehran Iran; ^7^ Student Research Committee Tabriz University of Medical Sciences Tabriz Iran

**Keywords:** CoQ10, glycemic indices, insulin, randomised controlled trials, type 2 diabetes, umbrella meta‐analysis

## Abstract

**Background:**

Several meta‐analyses suggest that Coenzyme Q10 (CoQ10) supplementation is associated with glycemic control; however, findings about fasting blood glucose (FBG) and haemoglobin A1c (HbA1c) remain inconsistent across studies. Accordingly, this study aimed to synthesise the results to present a firm conclusion in relation to the efficacy of CoQ10 on glycemic control.

**Methods:**

A systematic search was conducted to find meta‐analyses of randomised controlled trials using PubMed, Scopus, Web of Science and the Cochrane Database of Systematic Reviews from inception to March 6, 2025. Also, the methodological quality of included studies was evaluated using the AMSTAR2 tool.

**Results:**

In total, eight meta‐analyses were included in this umbrella systematic review and meta‐analysis. Pooled analysis using standardized mean difference analysis demonstrated that CoQ10 is associated with decreased FBG. While it didn't exert any significant changes on the HbA1c, HOMA‐IR, and insulin levels. In addition, the combined effect of CoQ10 using weighted mean difference analysis revealed that CoQ10 is able to decrease the FBG (5.04 mg/dL), HbA1c (0.17%), HOMA‐IR (0.72), and insulin (1.32 μIU/mL) levels significantly.

**Conclusion:**

The present study suggests that CoQ10 supplementation may have a moderate beneficial effect on glycemic control in diabetic patients, though findings differ depending on analytic approach.

## Graphical abstract

This umbrella review of meta‐analyses of randomised controlled trials shows that coenzyme Q10 supplementation modestly improves glycemic control, particularly fasting blood glucose. However, effects on HbA1c, insulin, and HOMA‐IR vary depending on the analytical approach used.

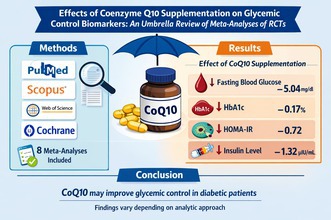

## 1 Introduction

Glycemic control has been associated with the development of several diseases. Evidence indicates that dysregulation of glucose metabolism makes individuals more susceptible to chronic diseases such as diabetes [1], obesity [2], dyslipidemia [3], and cardiovascular diseases (CVD) [4]. Optimal blood sugar control, essential for diabetes management, involves maintaining glycosylated haemoglobin (A1C) levels below 7.0%, as levels exceeding this threshold are associated with a significantly increased risk of microvascular and cardiovascular complications [4, 5].

In recent years, the intersection of nutraceutical supplementation and glycemic control has become a focal point in the quest for effective strategies to manage metabolic health [6]. Coenzyme Q10 (CoQ10), also known as ubiquinone, a vital component in the electron transport chain and a potent antioxidant, has garnered attention for its potential impact on various aspects of human health, including its hypothesized role in glycemic regulation [7, 8]. Furthermore, CoQ10 supplementation has been shown to exert cardioprotective effects in patients with heart failure [9].

CoQ10 is a fat‐soluble antioxidant substance widely found in living cells, which can effectively neutralise free radicals and protect cells from oxidative damage [10]. Recent research shows that people with type 2 diabetes (T2DM) have significantly lower levels of CoQ10 compared to their healthy counterparts [11, 12]. It has been shown that poor dietary intake [13], statin drug [14, 15], oxidative stress induced by hyperglycemia [16, 17, 18], insufficient absorption and also decrease in androgen production may contribute to decreased level of plasma CoQ10 [19]. Accordingly, CoQ10 supplementation may restore depleted levels and exert beneficial effects on related metabolic outcomes.

Several studies have supported the effectiveness of CoQ10 supplementation in improving fasting blood glucose (FBG), HOMA‐IR, and A1C [20, 21, 22]. The suggestion has been made that supplementing with external CoQ10 could serve as a potential strategy to alleviate mitochondrial dysfunction induced by oxidative stress, thereby improving FBG through this mechanism [23]. Furthermore, supplement of CoQ10 might elevate the function of tyrosine kinase, phosphatidylinositol kinase, and adiponectin receptors in diabetic mice while simultaneously reducing the activity of insulin receptor isoforms and glucose transporters [22].

Some meta‐analyses of randomised controlled trials (RCTs) have been conducted to assess the influence of CoQ10 on glycemic control, yielding contradictory outcomes [20, 24, 25, 26]. Hence, this present investigation was structured as an umbrella meta‐analysis to scrutinise the consolidated outcomes of CoQ10 supplementation on glycemic control biomarkers, as identified in prior meta‐analyses. The primary objective is to reconcile the disparities observed in the existing evidence.

## 2 Materials and Methods

This review adhered to the guidelines set by the Preferred Reporting Items for Systematic Reviews and Meta‐Analyses (PRISMA) [27], and we followed the instructions provided by the Joanna Briggs Institute (JBI) for performing the umbrella review [28]. Our protocol was registered in the Prospective Register of Systematic Reviews (PROSPERO) under CRD420251276572.

### 2.1 Search Strategy

Searched electronic databases including PubMed, Embase, Web of Science, and Cochrane were searched to collect clinical studies related to the efficacy of Q10 supplementation in the management of glycemic indices from inception to March 6, 2025 articles published in peer‐reviewed journals, using search strategy based on previous systematic reviews. Search is conducted by combining subjects and free words. Search terms and keywords used included combinations and variations of “coenzyme Q10” OR “CoQ 10” OR “ubidecarenone” OR “ubiquinone Q10” AND “Meta‐analysis”.

### 2.2 Inclusion and Exclusion Criteria

The PICO criteria for the present umbrella meta‐analysis were structured as follows: Population/Patients (P), This umbrella review included meta‐analyses of RCTs whose primary studies enrolled adult participants (≥ 18 years) with various baseline health conditions. As this is an umbrella review of existing meta‐analyses, the included populations are those from the constituent RCTs of the eight qualifying meta‐analyses. These populations, detailed in Table 1, were heterogeneous and included individuals with: T2DM, obesity/overweight, polycystic ovary syndrome (PCOS), chronic kidney disease (CKD: including those on haemodialysis), metabolic syndrome, and other cardiometabolic conditions. Participants in the primary RCTs were receiving various standard‐of‐care background treatments relevant to their condition (e.g., antidiabetic medications, statins, antihypertensives). The control groups in these RCTs received a placebo or continued their standard care, allowing for the assessment of the additive effect of CoQ10 supplementation. Outcome (O) glycemic control biomarkers including FBG, HbA1c, HOMA‐IR, and insulin. Only meta‐analysis studies published in English that explored the impact of CoQ10 supplementation on glycemic indices and had reported effect sizes (ES) along with their corresponding confidence intervals (CI) were considered for inclusion. Original studies, editorials, letters to the editor, children studies, and studies of pregnant or lactating women were excluded from consideration.

**TABLE 1:** Study characteristics of included studies.

Citation (first author et al., Year)	Location	Study population	Number of included studies	Sample size	Gender	Mean age	Q_10_ dosage (mg)	Duration (week)	Main outcome
Zhang et al., 2019	China	Diabetic kidney disease	8	135	Both	NR	376.6	NR	FBG↓ A1C↓
Zhang et al., 2023	China	PCO	9	353	Female	NR	200	8–9	FBG↓ HOMA‐IR↓
Huang et al., 2018	China	Overweight and obese patients with T2DM	14	693	Both	55–58.77	130–192.3	11.2–13	FBG↓ A1C↓ HOMA‐IR↔
Dludla et al., 2020	South Africa	Diabetic haemodialysis‐DN‐T2DM‐overweight and obese‐DM‐Mets	12	650	Both	54.59–56.9	144–185.6	10.66	FBG↔ A1C↓ Insulin↔
Bakhshayeshkaram et al., 2018	Iran	CKD‐HD‐ESRD‐DN	7	721	Both	57.9–58.9	110–150	10–10.66	FBG↔ HOMA‐IR↔ Insulin↔
Moradi et al., 2016	Iran	T2DM‐CAD‐CKD‐HTN‐T2DM and Dyslipidemia	16	920	Both	NR	188.2–200	9–14.81	FBG↓ A1C↔ Insulin↔
Liang et al., 2022	China	T2DM‐NAFLD‐dyslipidemia‐PCOS‐obesity‐CKD‐healthy	40	2424	Both	48.84–53.83	157.91–221.81	10.66–13.03	FBG↓ A1C↓ HOMA‐IR↓ Insulin↓
Stojanovic et al., 2021	Serbia	2D‐T1DM‐obesity‐CKD‐CAD‐HTN	18	820	Both	50.65–55.76	165.29–187.5	10.82–13.33	FBG↓ A1C↔

Abbreviations: CAD, coronary artery disease; CVD, Cardiovascular disease; DN, Diabetic nephropathy; ESRD, End‐Stage Renal Disease; HD, haemodialysis; HTN, *hypertension*; MetS, Metabolic Syndrome; NAFLD, nonalcoholic fatty liver disease; NR, not reported; PCO, Polycystic ovary syndrome; RA, Rheumatoid arthritis; T2DM, Type 2 diabetes.

### 2.3 Evaluating Methodology

Two researchers (PP and MJ) independently assessed the included studies' methodological quality using the AMSTAR2 evaluation tool [29], which includes 16 criterion heading towards a “yes”, “partial yes”, “no”, or “no meta‐analysis” responses. Any disagreements were resolved by mutual consensus or through the intervention of a third author (VM) if required.

### 2.4 Study Selection and Data Extraction

Studies were independently screened and data from the identified studies were extracted by two reviewers (PP and MJ) based on pre‐established criteria. This included the author's name, year of publication, sample size, study location, CoQ10 supplementation dosage and duration, ES and CI for glycemic indices.

### 2.5 Data Synthesis and Statistical Analysis

ES and CI calculated for each study were used to estimate overall effects. The presence of heterogeneity was evaluated using the *I* ^2^ statistic and the Cochrane *Q* test, considering significant heterogeneity when the *I* ^2^ value was > 50% or *p* < 0.1. In the presence of significant between‐study heterogeneity, pooled estimates were calculated using a random‐effects model based on the DerSimonian–Laird method. Subgroup analyses were also done to explore potential sources of heterogeneity. However, the small number of included meta‐analyses for some outcomes such as (e.g., only two meta‐analyses reporting SMD for HOMA‐IR) limited the ability to perform meta‐regression or subgroup analyses. Due to the natural differences between SMD and WMD, the analysis was performed for each separately. A one‐study removal sensitivity analysis was used to detect the dependency of the overall ES on a particular meta‐analysis. No small study effect was performed for any of the outcomes as none of them included at least 10 studies [30]. All statistical analyses were carried out using Stata software (version 16, Stata Corp., College Station, TX, US), considering a *p*‐value of < 0.05 significant. Although statistical significance was defined as a *p*‐value < 0.05, the interpretation of findings primarily emphasised the magnitude and precision of effect sizes rather than reliance on *p*‐values alone.

## 3 Result

### 3.1 Selected Studies and Systematic Review

**FIGURE 1:**
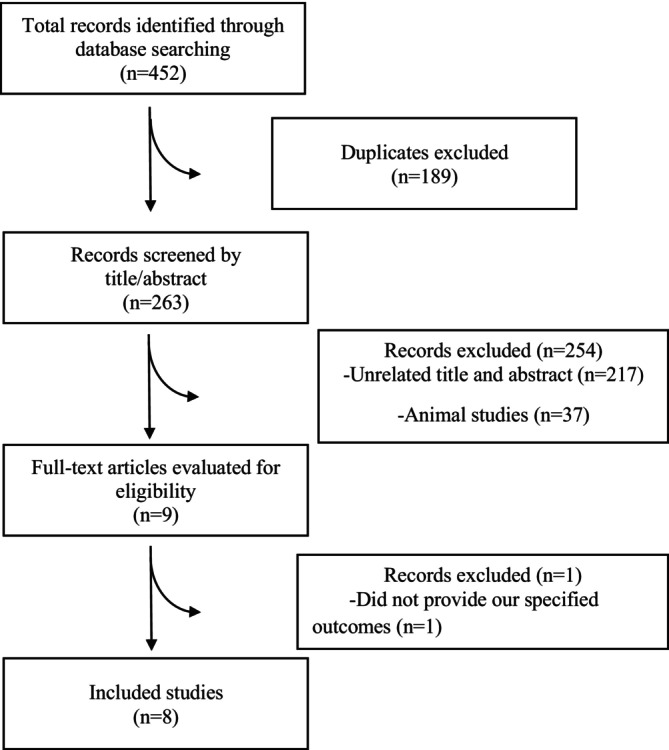
Flow diagram of study selection.

The literature search process, as illustrated in Figure 1 using the PRISMA flow chart, involved an initial identification of 452 articles through electronic database searches. Among these, 189 duplicates were identified and removed. After scrutinising the titles and abstracts of the remaining 263 studies, 254 articles were excluded as they did not meet the inclusion criteria. Consequently, eight meta‐analyses, published between 2016 and 2023, met the criteria for inclusion in the umbrella review. Table 1 presents the characteristics of these included meta‐analyses. The studies revealed an average administered amount of CoQ10 ranging from 110 to 376.6 mg/day, with CoQ10 supplementation durations spanning 8 to 14.81 weeks. Geographically, the studies were conducted in four locations: four in China [20, 21, 22, 31], two in Iran [24, 25], one in Serbia [26], and one in South Africa [32]. Table 2 displays the outcomes of the AMSTAR2 questionnaire. Eight of the articles demonstrated high quality, while one was categorised as moderate quality. First domain Q1 (protocol) of AMSTAR2 tool score as “No” for most of the included studies, which affects the overall score. In addition, funnel plots for study outcomes are provided in Supplementary file.

**TABLE 2:** Results of assessing the methodological quality of meta‐analysis.

Study	Q1	Q2	Q3	Q4	Q5	Q6	Q7	Q8	Q9	Q10	Q11	Q12	Q13	Q14	Q15	Q16	Quality assessment
Zhang et al., 2019	No	Partial yes	Yes	Partial Yes	Yes	Yes	Yes	Yes	Yes	No	Yes	Yes	Yes	Yes	Yes	Yes	High
Zhang et al., 2023	No	Partial yes	Yes	Partial Yes	Yes	Yes	Yes	Yes	Yes	No	Yes	Yes	Yes	No	Yes	Yes	Moderate
Huang et al., 2018	Yes	Yes	Yes	Yes	Yes	Yes	Yes	Yes	Yes	Yes	Yes	Yes	Yes	Yes	Yes	Yes	High
Dludla et al., 2020	No	Yes	Yes	Partial Yes	Yes	Yes	Yes	Yes	Yes	Yes	Yes	Yes	Yes	No	Yes	Yes	High
Bakhshayeshkaram et al., 2018	No	Partial yes	Yes	Partial Yes	Yes	Yes	Yes	Yes	Yes	Yes	Yes	Yes	Yes	Yes	Yes	Yes	High
Moradi et al., 2016	No	Partial yes	Yes	Yes	Yes	Yes	Yes	Yes	Yes	No	Yes	Yes	Yes	Yes	Yes	Yes	High
Liang et al., 2022	No	Partial yes	Yes	Yes	Yes	Yes	Yes	Yes	Yes	No	Yes	Yes	Yes	Yes	Yes	Yes	High
Stojanovic et al., 2021	No	Partial yes	Yes	Partial Yes	Yes	Yes	Yes	Yes	Yes	Yes	Yes	Yes	Yes	Yes	Yes	Yes	High

### 3.2 Effect of CoQ10 on FBG According to SMD, and WMD Analysis

**FIGURE 2:**
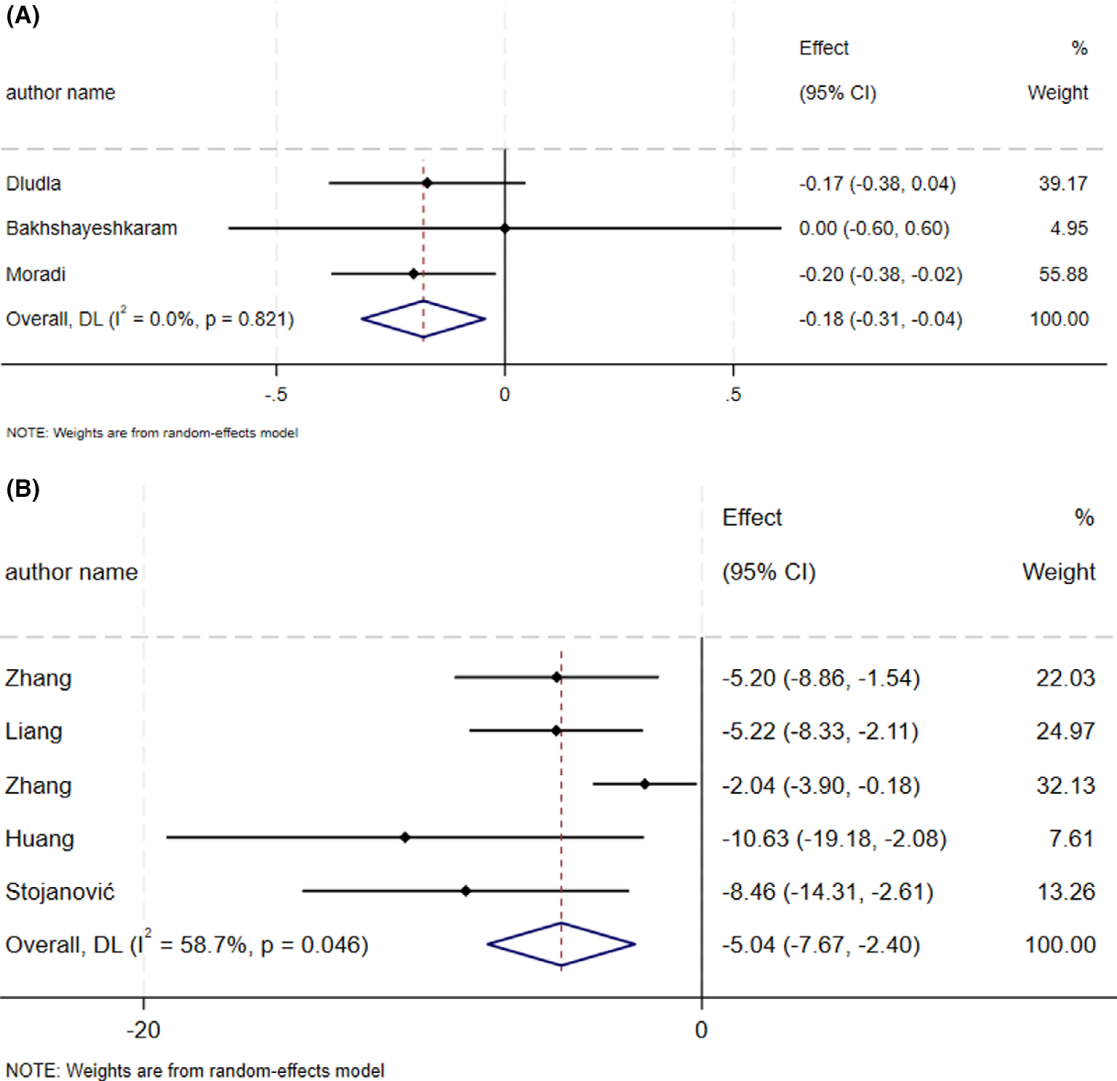
Forest plot detailing effect size and 95% confidence intervals (CIs), the effects of CoQ10 supplementation on FBG levels according to SMD analysis (A), and WMD analysis (B).

The supplementation of CoQ10 led to a notable decrease in FBG levels (ES_SMD_ = −0.18, 95% CI: −0.31, −0.04; *p* = 0.009, *I* ^2^ = 0.0%, *p* = 0.82) (Figure 2A) [24, 25, 32]. The administration of CoQ10 resulted in a significant decrease in FBG levels (ES_WMD_ = −5.04, 95% CI: −7.67, −2.40; *p* < 0.001, *I* ^2^ = 58.7%, *p* = 0.04) (Figure 2B) [20, 21, 22, 26, 31]. Conducting sensitivity analysis did not reveal any notable distinctions.

### 3.3 Effect of CoQ10 on HbA1c According to SMD, and WMD Analysis

**FIGURE 3:**
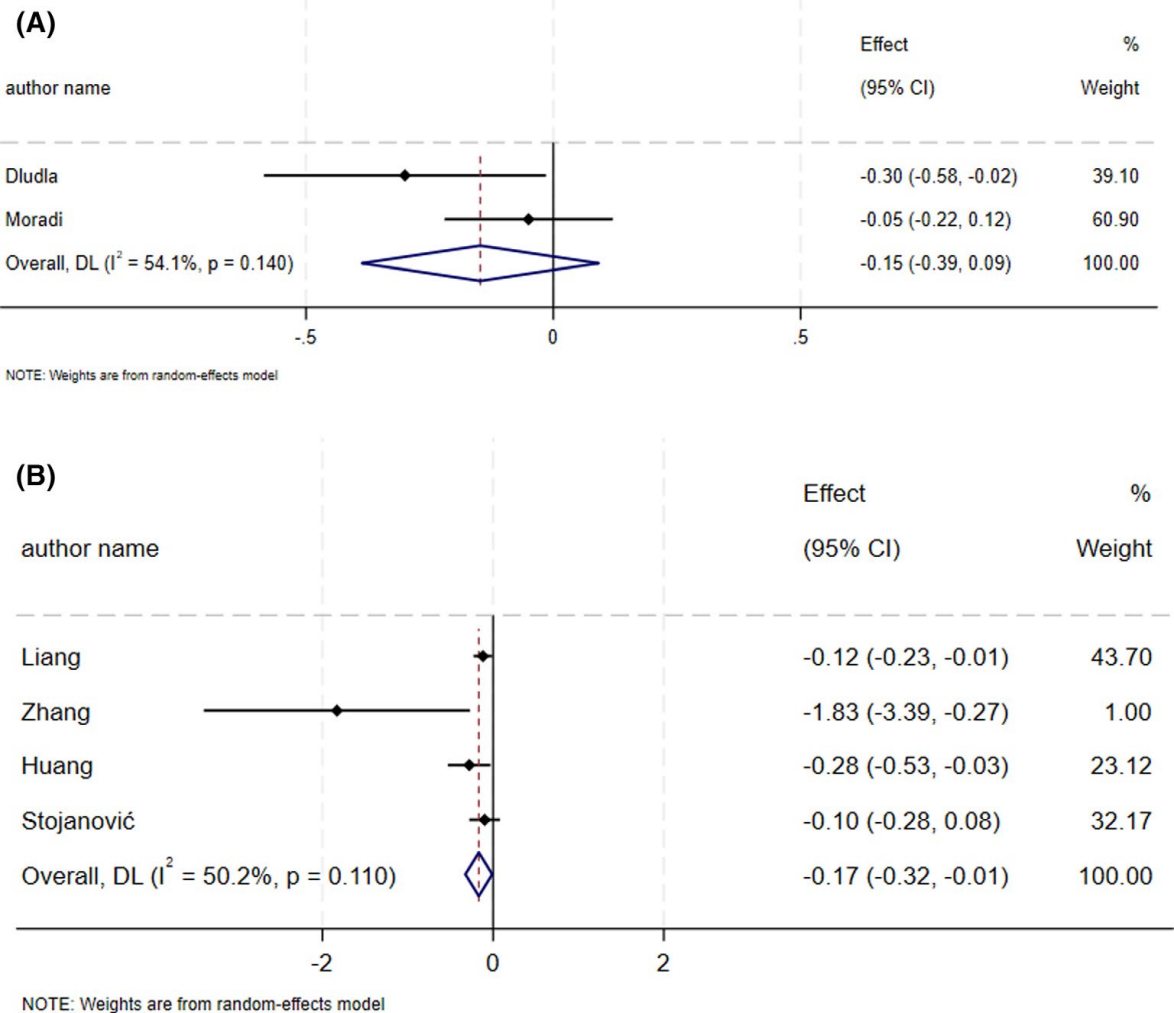
Forest plot detailing effect size and 95% confidence intervals (CIs), the effects of CoQ10 supplementation on HbA1c levels according to SMD analysis (A), and WMD analysis (B).

The supplementation of CoQ10 did not result in a significant influence on HbA1c levels (ES_SMD_ = −0.15, 95% CI: −0.39, 0.09; *p* = 0.22, *I* ^2^ = 54.1%, *p* = 0.14) (Figure 3A) [25, 32]. CoQ10 supplementation resulted in a significant decrease in HbA1c levels (ES_WMD_ = −0.17, 95% CI: −0.32, −0.01; *p* = 0.03, *I* ^2^ = 50.2%, *p* = 0.11) (Figure 3B) [21, 22, 26, 31]. Sensitivity analysis showed that after removing any of the three studies [21, 22, 26], the result changed non‐significantly.

### 3.4 Effect of CoQ10 on HOMA‐IR According to SMD, and WMD Analysis

**FIGURE 4:**
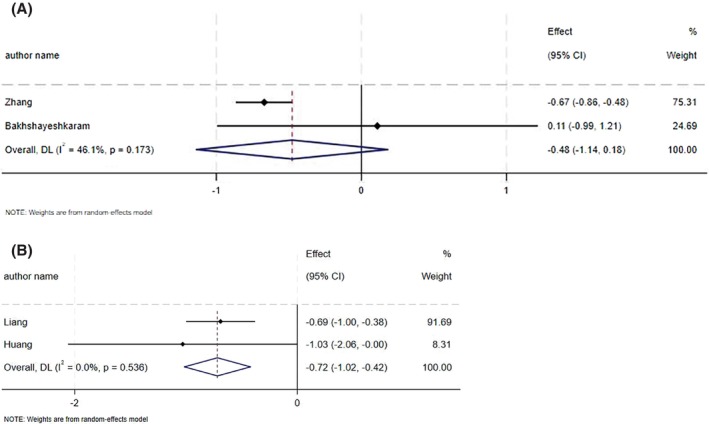
Forest plot detailing effect size and 95% confidence intervals (CIs), the effects of CoQ10 supplementation on HOMA‐IR levels according to SMD analysis (A), and WMD analysis (B).

CoQ10 supplementation had no significant impact on HOMA‐IR (ES_SMD_ = −0.48, 95% CI: −1.14, 0.18; *p* = 0.15, *I* ^2^ = 46.1%, *p* = 0.17) (Figure 4A) [20, 24]. CoQ10 led to a significant decrease in HOMA‐IR (ES_WMD_ = −0.72, 95% CI: −1.02, −0.42; *p* < 0.001, *I* ^2^ = 0.0%, *p* = 0.53) (Figure 4B) [21, 22].

### 3.5 Effect of CoQ10 on Insulin According to SMD, and WMD Analysis

**FIGURE 5:**
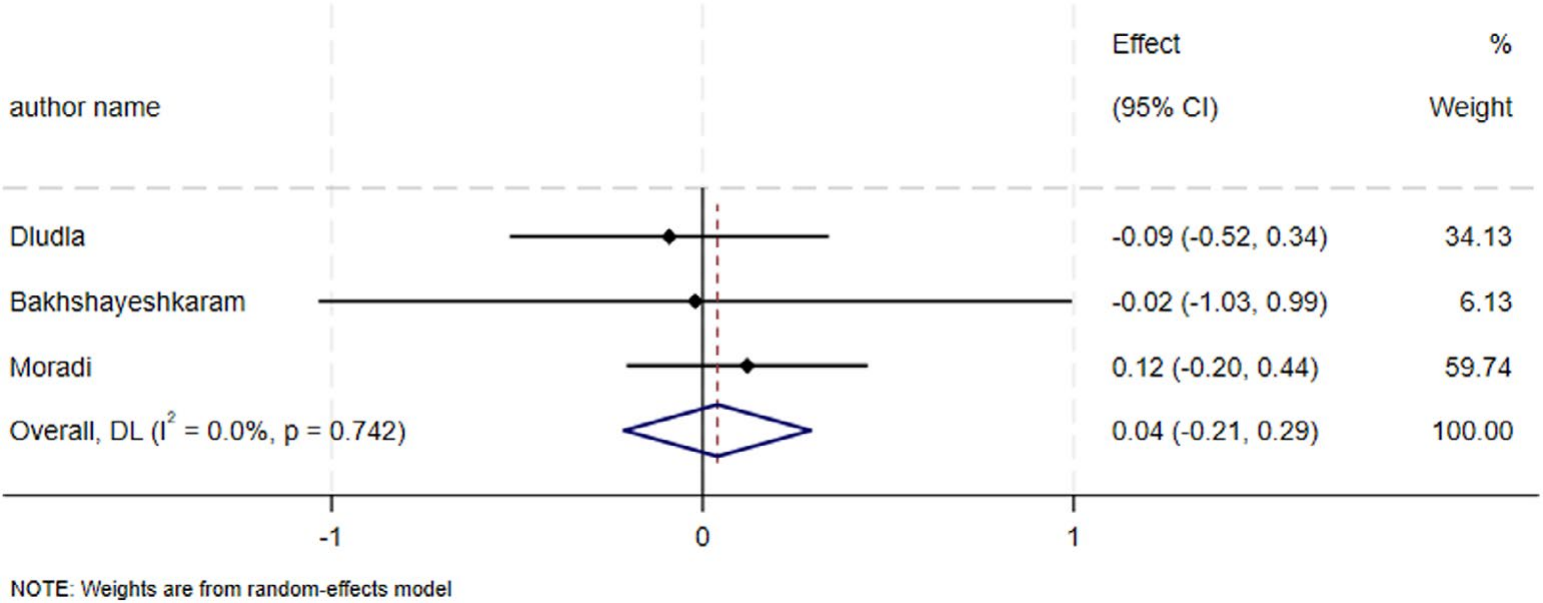
Forest plot detailing effect size and 95% confidence intervals (CIs), the effects of CoQ10 supplementation on insulin levels according to SMD analysis.

Administration of CoQ10 did not result in a significant effect on insulin levels (ES_SMD_ = 0.04, 95% CI: −0.21, 0.29; *p* = 0.75, *I* ^2^ = 0.0%, *p* = 0.74) (Figure 5). The administration of CoQ10 led to a notable reduction in insulin levels (ES_WMD_ = −1.32, 95% CI: −2.06, −0.58; *p* < 0.001).

## 4 Discussion

Based on the results obtained from WMD analyses, supplementation with CoQ10 significantly reduces HbA1c, FBG, and HOMA IR. Also, based on SMD analysis, CoQ10 significantly reduces FBG, but it has no significant effect on HbA1c, HOMA IR, and insulin level.

There are different mechanisms that explain the effect of CoQ10 on the changes of the mentioned outcomes: Antioxidant Activity: CoQ10 is a potent antioxidant that helps neutralise free radicals and reduce oxidative stress. Oxidative stress plays a role in the development of insulin resistance and impaired glucose metabolism. By reducing oxidative stress, CoQ10 may help improve insulin sensitivity and glycemic control, leading to a potential decrease in HbA1c, HOMA IR, and FBG [11, 33]. Cellular Energy Metabolism: CoQ10 is an essential part of the electron transport chain in mitochondria, where energy is made. CoQ10 supplements may help the body use glucose better and respond better to insulin by improving mitochondrial function and cellular energy production. This function could lead to decreased HbA1c, HOMA IR, and FBG [34, 35, 36]. Inflammation Modulation: Insulin resistance and problems with glucose metabolism are linked to chronic low‐grade inflammation. CoQ10 can reduce inflammation by stopping the production of proteins that cause inflammation. By lowering inflammation, taking extra CoQ10 may improve insulin function and help control blood sugar, which could affect HbA1c, HOMA IR, and FBG levels [37]. Beta‐cell Function: It has been suggested that supplemental CoQ10 can help the pancreatic beta cells, making insulin work better and stay alive. CoQ10 may help keep insulin levels regular and improve glucose balance by protecting the health and function of beta cells [11, 38, 39].

As mentioned, based on SMD analysis, CoQ10 supplementation had no significant effect on HbA1c, HOMA IR, and insulin levels. In the case of HbA1c and HOMA IR, the small number of studies included is a possible reason for this issue. In the case of insulin levels, the low sample size of the included studies is the reason for the non‐significance of the overall result. Based on SMD analysis and Regarding the HOMA IR outcome, two studies, Bakhshayeshkaram and Zhang, have been entered [20, 24]; the result of Bakhshayeshkaram study is not significant, which is the possible reason for its small sample size compared to Zhang study. Regarding the FBG outcome and based on the SMD analysis, the overall result shows a significant reduction in FBG. However, the result of the study by Bakhshayeshkaram et al. [24]. was not significant, which is a possible reason that the average intervention dosage has been lower and the sample size smaller than in other studies. The results of the WMD analysis showed that CoQ10 can significantly reduce FBG. However, the study of Stojanović et al. [26]. shows the opposite of the general result. As in the previous case, the sample size and lower supplemental dose (< 200 mg) are the possibly reasons for this issue. The same applies to HOMA IR outcome (WMD analysis), and the study by Huang et al. [21]. obtained a non‐significant result due to the low sample size, which conflicts with the general result. Based on the results, the study of Huang et al. [21]. has had the greatest impact on reducing FBG. The effect size of this study is twice that of other studies. One of the reasons for this is the target group of this study, which includes obese and overweight people as well as people with diabetes. For the following reasons, CoQ10 supplementation has a greater effect on the improvement of FBG in these people: Insulin Resistance: Insulin resistance happens when cells stop responding to insulin. Being overweight or obese is often linked to this disease. Supplementary CoQ10 can make insulin work more effectively, which could help overweight and obese individuals take in and use glucose better, lowering their FBG levels even more [23]. Oxidative Stress: Obese and overweight individuals often experience increased oxidative stress due to the production of excess reactive oxygen species. CoQ10's antioxidant properties may help alleviate oxidative stress, which can contribute to improved insulin sensitivity and glucose metabolism, resulting in a greater impact on glycemic control [11, 40]. Inflammation: Obesity is characterised by chronic low‐grade inflammation, which can impair insulin signalling and lead to insulin resistance. CoQ10 has been shown to possess anti‐inflammatory properties, potentially reducing inflammation in obese individuals and improving insulin sensitivity, thus contributing to a greater reduction in glycemic index [41, 42]. Impaired Insulin Secretion and Increased Insulin Resistance: Individuals with diabetes often have trouble secreting insulin and are more resistant to insulin, which causes their blood sugar levels to rise. CoQ10 may help improve insulin production and insulin sensitivity, which can help people with diabetes better control their blood sugar [11, 43]. Medication Interactions: To keep their blood sugar levels in check, people with diabetes often take oral hypoglycemic drugs or insulin. Taking supplemental CoQ10 might help lessen the harmful effects of some diabetes drugs, like statins, which can lower the amount of CoQ10 in the body. By restoring CoQ10 levels, supplements help control blood sugar better and lessen the side effects of medications [11, 25].

## 5 Strength and Limitation

Based on the searches, the current study is the first meta‐analysis study on the effect of CoQ10 on glycemic indices. Also, this study was registered in Prospero. In this study, all relevant indicators have been analysed, and accurate and reliable results have been obtained. First, one of the limitations of our study is the low number of included articles, which made it impossible to perform meta‐regression and subgroup analyses and more accurate and exciting results. Second, limited number of included studies contributed to restricted us to perform statistical tests for publication bias. However, possibility of publication bias cannot be ignored. The inclusion of overlapping primary studies across multiple meta‐analyses posed a potential concern; however, our assessments indicated that this overlap had minimal influence on the overall findings. Third, we have restricted to English‐publications which may lead to language bias. Fourth, most of the included studies are conducted in Asian which limits the generalizability of our findings. Fifth, the participant populations across the included meta‐analyses were clinically heterogeneous, encompassing individuals with different metabolic disorders (e.g., T2DM, PCOS, CKD) who were likely on varied background pharmacotherapies. This heterogeneity may influence the magnitude and consistency of CoQ10's effect on glycemic parameters and is an important context for interpreting our pooled findings. The interaction between CoQ10 and specific antidiabetic or other medications remains an area for further research.

## 6 Conclusion

In the end, as a general conclusion, it can be said that supplementation by CoQ10 can improve glycemic control biomarkers, especially FBG, in people with different health conditions such as those with obesity, overweight, or various diseases.

## Author Contributions

V.M. and M.M. designed research; M.J., M.F. and V.M. conducted systematic search; E.S. and P.P. screened articles, extracted data and drew tables; V.M. analysed and interpreted data; M.M., V.M., F.S. and M.F. wrote the paper. V.M., and F.S. had primary responsibility for final content. All authors read and approved the final manuscript.

## Funding

The authors have nothing to report.

## Ethics Statement

The authors have nothing to report.

## Consent

The authors have nothing to report.

## Conflicts of Interest

The authors declare no conflicts of interest.

## Data Availability

The data that support the findings of this study are available from the corresponding author upon reasonable request.
